# A History of the Inductive Sciences

**Published:** 1838-04

**Authors:** 


THE
### BRITISH AND FOREIGN
### MEDICAL REVIEW,

FOR APRIL, 1838.

### PART FIRST.

### Analytical and Critical Reviews.

### Art. I.

*A History of the Inductive Sciences.* By the Rev. W. Whewell, m.a. Fellow and Tutor of Trinity College, Cambridge; President of the Geological Society, &c.—*London*, 1837. Three Vols. 8vo. pp. 437, 534, 624.

The time, we would fain hope, is now gone by when attainments in knowledge of every kind, not immediately connected with medical practice, were looked on by the public, and even by many of the profession, with a suspicious eye; and when the man of science, who devoted his leisure hours to the combination of amusement and instruction afforded by his laboratory or his study, was thought on that account less worthy of confidence than the frivolous partaker in public gaieties. A better feeling on this subject is gradually gaining ground; and we could point to many distinguished individuals who have shewn that scientific pursuits are by no means incompatible with professional eminence and reputation. The extended course of education now required by our corporate bodies is of itself sufficient to impart no trifling amount of general knowledge to those who fully avail themselves of its opportunities; and, knowing how important is the influence of our profession on the tone of society at large, and how much it has the means of improving the taste for knowledge among all classes of the community, we cannot but anticipate a still farther change in the estimate formed by the public of its requirements.

We do not urge the attainment of general scientific knowledge merely as a matter of expediency: we deem an acquaintance with the principles of physical science essential to every one who is not content with treading the beaten path of medical practice, and who cherishes the higher object of extending its boundaries and diminishing its perplexities. We well remember to have heard it remarked, by one whose long life has been spent in the acquirement and diffusion of professional knowledge, that, when physiology and pathology should be disencumbered of theories which are physically impossible, there would be some hope of their advancement. It is, in fact, only by the accurate separation of physical from vital actions in the living system that we can expect to arrive at any such knowledge of the latter as will enable us to classify and generalize the phenomena presented to our notice. No one can attempt this, with any prospect of success, who is not conversant with the laws of inorganic matter, and who is not prepared to carry his conception of their application to an extent still wider than he can discern from the eminence they have hitherto attained. In proportion to the elevation and comprehensiveness of our generalizations, do we find the boundaries which at present divide the sciences disappear; just as the aeronaut, in enlarging his horizon, successively loses sight of the divisions which the art of man or the hand of nature has interposed to separate from each other, estates, provinces and kingdoms.

It is quite evident to every discriminating enquirer, that the phenomena of which the sum constitutes what we know of life, are generally of a very complex nature; that certain vital properties peculiar to organized structures are essentially concerned in the production of most of them; and that the common physical properties of matter are also frequently brought into operation: but that there is a class of actions, in the production of which both physical and vital properties partake, and in the analysis of which there is, consequently, great difficulty. It is, in fact, one of the most important problems in physiology, to determine how far the results of physical laws can be modified by the conditions supplied by the operations of vitality; and it requires a mind thoroughly imbued, not only with the principles of general science, but also with that philosophical spirit which alone can render the knowledge of them available, to pursue the enquiry with the probability of success.

It is not one of the least among the advantages afforded by the cultivation of general science, that, if properly directed, it exercises a beneficial influence on those faculties of the mind, the training of which is essential to the successful pursuit of medical knowledge. The same acuteness of observation, the same cautiousness of inference, the same discrimination in classifying facts, the same sagacity in generalizing them, and the same skill in developing the application of such generalizations, are required in the sciences of life as in those of inert matter.

It is far from our wish to encourage that discursive spirit, which, by directing its attention to too many objects at the same time, is unable to gain a distinct view of any. We feel convinced that, when once fully engaged in the practice of his profession, no one has a right to devote any considerable portion of his time to extraneous pursuits: but even then, if a good foundation have been laid in earlier life, we believe it to be in the power of every one to keep pace with the attainment of laws, and to acquire the knowledge of their application, not only in one or two departments of science, but in all to which his attention has originally been directed. For, be it remembered that every step in generalization is in reality an advance in simplification, and that every law is the comprehensive expression of a vast number of facts, which had previously to be individually retained in the memory; so that, whilst the ingenuity and industry of scientific enquirers are constantly developing new facts, and thus apparently increasing the complexity and extent of each branch of knowledge, these very facts, combined by the talents of the philosopher with others previously ascertained, conduct him to the discovery of general laws, which include not only the phenomena upon which they were based, but others previously supposed to be beyond their pale, and which lead, by simple deductive reasoning, to results hitherto unexpected.*

To those who agree with the views expressed in the preceding remarks we can strongly recommend the work of Mr. Whewell, as affording much instruction in a useful and attractive form. A mere history of any science is to most persons a "flat, stale, and unprofitable" performance; but, in proportion as it developes the origin, progress, and effects of successive doctrines, the bearing of successive discoveries on the subsequent attempts at generalization,—in short, in proportion as it becomes a philosophical history, does it become generally interesting and valuable. The object proposed by Mr. Whewell is to point out the consecutive steps by which the different sciences have arrived, by the process of induction, at the respective elevations which they have already attained; and, keeping this definite end in view, he necessarily disregards all the events which have no immediate bearing upon it. As there have been few systems of philosophy, however, without some foundation in truth, (most of them being the result of a too-rapid generalization of a limited number of facts,) each one of these has brought prominently forward a certain amount of knowledge, which has in the end been serviceable towards the perfection of science. Hence, in such a history as we are considering, the hypothetical dogmas which have at different times exercised a powerful sway over the human intellect, are not overlooked; and the most correct estimate of the value of each may be formed by considering the effect which it has had in widening the legitimate boundaries of knowledge, and in contributing towards the establishment of the truth by the overthrow of other systems still more erroneous. The mode in which the writer has executed the task he has proposed for himself, is such as might be expected from the distinguished station which he holds among the cultivators of physical science. If we should hereafter think it necessary freely to express an opinion to the disadvantage of the portion dedicated to Physiology, we shall do it with much regret that an author of so high and deserved a reputation should have in any degree disparaged it by venturing to treat of the philosophy of a subject with whose details he is not conversant. We think that Mr. Whewell need not have gone beyond the limits of his own university in search of a writer fully competent to assist him in his plan; and we cannot but believe that, although a single mind may carry into such a work more uniformity of design than could

* We are happy in being able to strengthen our remarks on this subject by the authority of M. Magendie, who says, in one of his recent lectures, "I am so strongly convinced of the necessity of a knowledge of physical science for the comprehension and practice of medical art, that I cannot too strongly urge the study upon you. Not that I pretend that a man cannot be a good practitioner without the acquirements of MM. Thenard, Arago, Poisson, &c.; but I maintain, that you ought not to be unacquainted with the *principles* of the exact sciences. They alone can unveil a mass of phenomena, of which the operation would otherwise be inexplicable; they alone can assist the science of medicine in freeing itself from the trammels in which it has been confined by ignorance and the mania of systematising."—*Leçons sur les Phénomènes Physiques de la Vie*, tome ii. p. 27.

be possessed by a plurality, there must, of necessity, be great inequalities in the execution.

Before entering upon this particular topic, however, we shall briefly follow our author through the subjects of his first two volumes, which are devoted to the exact sciences; and we hope that our statements will not be considered irrelevant to the ultimate object of the present article. Our first quotation shall be from the general introduction, which contains some thoughts peculiarly applicable, as it appears to us, to the study of physiology.

"To the formation of science two things are requisite: facts and ideas; observations of things without, and an inward effort of thought; or, in other words, sense and reason. Neither of these elements, by itself, can constitute substantial general knowledge. The impressions of sense, unconnected by some rational and speculative principle, can only end in a practical acquaintance with individual objects; the operations of the rational faculties, on the other hand, if allowed to go on without a constant reference to external things, can lead only to empty abstraction and barren ingenuity. Real speculative knowledge demands the combination of the two ingredients; right reason, and facts to reason upon. It has been well said, that true knowledge is the interpretation of nature; and thus it requires both the interpreting mind and nature for its subject; both the document and the ingenuity to read it aright. Thus, invention, acuteness, and connexion of thought are necessary, on the one hand, for the progress of philosophical knowledge; and, on the other hand, the precise and steady application of these faculties to facts well known and clearly conceived. It is easy to point to instances in which science has failed to advance in consequence of the absence of one or other of these requisites: indeed, by far the greater part of the course of the world, the history of most times and most countries, exhibits a condition thus stationary with regard to knowledge. The facts, the impressions on the senses, on which the first successful attempts at physical knowledge proceeded, were as well known long before the time when they were thus turned to account as at that period. The motions of the stars, and the effects of weight, were familiar to man before the rise of the Greek astronomy and mechanics; but the 'diviner mind' was still absent; the act of thought had not been exerted, by which these facts were bound together under the forms of laws and principles. And, even at this day, the tribes of uncivilized and half-civilized man over the face of the earth have before their eyes a vast body of facts of exactly the same nature as those with which Europe has built the stately fabric of her physical philosophy; but, in almost every other part of the earth, the process of the intellect by which these facts become science is unknown. The scientific faculty does not work. The scattered stones are indeed there, but the builder's hand is wanting. And, again, we have no lack of proof that mere activity of thought is equally inefficient in producing real knowledge. Almost the whole of the career of the Greek schools of philosophy, of the schoolmen of Europe in the middle ages, of the Arabian and Indian philosophers, shews us that we may have extreme ingenuity and subtilty, invention and connexion, demonstration and method; and that yet out of these germs no physical science may be developed. We may obtain by such means logic and metaphysics, and even geometry and algebra; but out of such materials we shall never form mechanics and optics, chemistry and physiology. How impossible is the formation of these sciences without a constant and careful reference to observation and experiment; how rapid and prosperous may be their progress when they draw from such sources the materials on which the mind of the philosopher employs itself; the history of those branches of knowledge for the last three hundred years abundantly teaches us." (Vol. i. p. 9.)

If, as we shall presently endeavour to shew, the true science of physiology is at present in its infancy, the ends to be attained not generally understood, and the most satisfactory means of pursuing them not fully determined, its cultivators cannot do better than take warning by the errors which the history of general philosophy brings prominently before them. We regard this science as at present slowly undergoing that revolution which Bacon, Galileo, and Newton concurred to effect in general physics; the ruins of exploded systems have clung so pertinaciously around it, that not even the vast intellect and unwearied perseverance of Haller could succeed in developing its true form and proportions; and, until it is entirely disencumbered of their remains, we do not expect that it will make any decided advance. Some passages, in the part of Mr. Whewell's work which relates to the physical philosophy of the Greeks, might be made, by a slight alteration in terms, equally applicable to the school of physiology which is but just passing away.

"The radical and fatal defect in the physical speculations of the Greek philosophical schools was, that, though they had in their possession facts and ideas, *the ideas were not distinct and appropriate to the facts.* There is no difficulty in perceiving that, for each class of facts, there is some special set of ideas, by means of which the facts can be included in general scientific truths; and that these ideas, which may thus be termed *appropriate,* must be possessed with entire distinctness and clearness, in order that they may be successfully applied. For example: one of the facts which Aristotle endeavours to explain is this,—that, when the sun's light passes through a hole, whatever be the form of the hole, the bright image, if formed at any considerable distance from the hole, is round, instead of imitating the figure of the hole, as shadows resemble their objects. We shall easily perceive this appearance to be a necessary consequence of the circular figure of the sun, if we conceive light to be diffused from the luminary by means of straight rays proceeding from every point. But, instead of this appropriate idea of rays, Aristotle attempts to explain the fact by saying that the sun's light has a *circular nature,* which it always tends to manifest. And this vague and loose conception of a circular quality, employed instead of the distinct conception of rays which is really applicable, prevented Aristotle from giving a true account even of this very simple optical phenomenon." (P. 81.)

The causes which thus retarded the progress of physical science among the Greeks have been perpetuated in physiology down to the present time. There are still men, aspiring to the title of philosophers, who are content to refer all the operations of life which they cannot explain by physical laws to the "Vital Principle;" a term which conveys about as definite an idea as the "circular nature" of Aristotle; instead of viewing them as the results of vital properties by whose action they are produced, (just as the physical properties of matter give rise to its physical changes,) and of which the characters and laws are as open to investigation as those of the more evident qualities, although more difficult of attainment owing to the complexity of the combinations in which the results are presented to our examination.

In considering the claim of physiology to be regarded as an inductive science, we shall take occasion to point out what we believe to be the legitimate method of pursuing it; since there are many who imagine that, in the investigation of vital and physical phenomena, entirely distinct courses are to be pursued, and comparatively few who are aware how similar are the tracks in both; the differences, which all perceive, affecting more the preparatory steps and arrangements than the actual object or direction of the journey. In our enquiry into the laws which regulate the complex phenomena of nature, (or, rather, the general expression of the conditions under which they occur,) our first object is to collect a sufficient number of instances, having an obvious relation to one another, with a view of determining the circumstances common to all. These instances are furnished to us by *observation;* and, where the phenomena are so simple that their antecedents are uniformly the same, they only need to be associated a sufficient number of times for the mind to be satisfied of the constancy of the relation. In general, however, even where a particular antecedent and consequent present such an invariable relation as necessarily excites the idea of causation, several concurrent circumstances influence the general result so far as, at first sight, to give it a degree of uncertainty; and the value and influence of each of these circumstances has to be determined before we can regard our knowledge of the conditions of the phenomena as complete. Still more frequently does it happen that, of several antecedent phenomena, it is difficult, or even impossible, to fix upon the single one which possesses an invariable relation with those which are the subjects of analysis. Here it is, therefore, that the process of the *artificial* production of phenomena, termed *experiment,** is so useful, by enabling us to make new combinations of the antecedents, in such a manner as to test every condition which can affect the general result. It is obvious that the information thus attainable will frequently be precisely that which is deficient in our previous collection of observations; and it will be found that, in proportion as any science affords facilities for thus "asking questions of nature," the enquirer in that particular department will have the greater probability of becoming acquainted with her secrets. Another circumstance on which the attainment of general principles in any branch of science greatly depends, is the facility or difficulty it presents in the comparison of phenomena. Where the changes, occurring in a number of individual instances, are so evidently similar in character as to imply an identity of cause, there is comparatively little difficulty in deducing the general law of the effects; but, in more complex instances, where the operation of the real cause is, as it were, masked by the influence of concurrent conditions, or where (as often happens in physiology) the effects of the same apparent cause are totally different according to the instruments through which it operates, it is obvious that there will be great difficulty in the first stage of the inductive process,—that of the classification of phenomena,—so great, indeed, that it may be regarded as one of the principal obstacles to the advancement of those branches of science in which it presents itself.

The phenomena of the equilibrium and motion of inorganic bodies, comprehended in the sciences of *Statics* and *Dynamics,* will illustrate the influence of facility in the comparison of instances and in the application of experiment, in leading to the knowledge of general laws. The principles which have been attained by these means are so comprehensive that a process of simple deduction enables us to predict or account for all the phenomena which can occur; the only difficulty under which philosophers still labour in regard to them arises from their ignorance of

* We are aware that this definition of *experiment* does not altogether conform either with its etymology or with the sense in which it is commonly used. It is, however, one which designates its strictly limited application in physical philosophy; and we shall hereafter point out the utility of drawing a broad line in physiological research between those cases in which it is our object to ascertain phenomena as they actually and normally occur, and those in which we endeavour to produce new subjects for observation by a change in the conditions of the phenomena.

the molecular constitution of bodies, which renders the application of these laws to liquid and aeriform media still somewhat uncertain and difficult. The laws of statics and dynamics may themselves be comprehended in still more general expressions, and all the truths of both sciences may be deduced from a single analytical formula, of whose application the law of gravity (the extension of which from terrestrial matter to the solar system, and more recently to the universe, constitutes the grandest illustration of the inductive philosophy,) is but a single instance.*

In Chemistry, too, the direct resemblance of certain groups of phenomena renders their classification (the preparatory step to induction) a matter of little difficulty; whilst, at the same time, the facility of experiment enables us to fill up the voids which observation alone would leave; so that the simple *quantitative* expression of which its facts are usually capable, and the variety of combinations of causes and conditions so easily obtained by experiment, have led to the discovery of laws of a high degree of generality, which are probably themselves subordinate to others yet to be discovered. It is a remarkable consequence of the rapidity of generalization which led to the establishment of the law of definite proportions, that "its subordinate laws,—those which limit its generality in particular cases, which diminish the number of combinations abstractedly possible, and restrain the indiscriminate mixture of elements,—remain to be discovered."† Since the researches of Faraday have proved the identity of chemical and electrical attraction, it would seem that the science of chemistry can scarcely be regarded as having a distinct existence, and that it must ultimately merge in those of electricity and dynamics, which are themselves probably to be included in a single general expression.

In the sciences of Light and Heat, it is remarkable that the most recent discoveries, and the general views founded upon them, seem to do away altogether with the idea of these principles as distinct, imponderable, but material agents. With respect to *light*, indeed, the fundamental doctrines of the undulatory theory may now be regarded as fully established; and it is only in their application to an immense mass of phenomena that any delay can take place, owing to the laborious nature of the process, and the intricate mathematical reasoning required. The undulatory theory of *heat* is found capable of answering all obvious objections, and may be regarded as on its trial, to be confirmed or modified by future discoveries, and especially by an enlarged knowledge of the laws of the polarization of heat.

The present condition of the science of Electricity is peculiarly interesting to the physiologist. The immense number of new and unexpected phenomena detected in recent times by the industry of experimental enquirers, have been made subservient to the discovery of general laws, which not only include the facts upon which they were founded, but others at first sight of an entirely different character. In this manner not only voltaic, atmospheric, and animal electricity have been proved to be but forms of common electricity; but even magnetic phenomena appear to result from a peculiar application of the same powers, or, in other words, a peculiar manifestation of the same properties. Here, then, we have effects of a

* Whewell, vol. ii. p. 121. † Herschell's Preliminary Discourse, p. 306.

most dissimilar character, occurring as the necessary results of a common cause operating under a variety of conditions; and we cannot but hope that a corresponding simplification may, at some future time, be effected in physiological science, by the comprehension of those vital properties which are at present our ultimate facts (and whose laws cannot be said to be yet established,) under one general expression. Moreover, the progress of discovery in electrical science, and other allied branches, renders it probable that electricity is no longer to be regarded as a distinct, material, though imponderable, agent, but that it is rather to be looked on as a general *property* of matter, like attraction of other kinds; and that the actions which we witness, and the powers which are developed, are nothing more than the manifestation of this property, according to the conditions in which the material bodies concerned in them are placed. "It cannot be denied," says Mr. Whewell, "that the theory of the electric fluids affords a plausible explanation of facts; but it may fairly be questioned whether it is *necessary*, and the analogy of light and heat (and especially the polarization of the latter,) has done much to shake the theory of the electric fluids as a physical reality." The most beautiful generalization, however, of which this branch of science seems capable is that recently proposed by Mossoti, who has rendered it highly probable that the phenomena of statics and dynamics, with those of the molecular actions of bodies, and those of all the forms of electric power, may be included under the same general expression.

If the views here stated as to the non-existence of a distinct electric fluid, and the possession of electric properties by all matter, should prove correct, we cannot but consider them as having a most important bearing on physiological speculation. Our analysis of the functions of living beings, or of those changes whose sum constitutes their life, terminates in referring them all to certain properties possessed by their component structures, excited to action by the relations in which they are placed to each other and to external agents. Some of these properties we know to be *physical,* since they are equally manifested by dead and living organized tissues; others are of a different character, being essentially *vital,* and necessarily implying the pre-existence of a living system, by which inorganic matter has undergone the process of organization, and in which alone these properties can be manifested. The existence of these vital properties must for the present be regarded as our ultimate facts in physiology; but we may reasonably enquire whence they are derived. It is commonly said that an organized body, in assimilating and organizing the nutrient matter, by which the changes necessary to its existence are maintained, communicates or superadds to it, at the same time, those vital properties of which it was itself previously possessed. A logical exception might be taken to this form of expression, since we can scarcely regard that as communicable which has not a distinct existence; and, if we understand the term *property* aright, it merely expresses the relation between matter, in some particular form or state, and the percipient mind. But, passing by this consideration, we may advantageously enquire into the analogy on which the view just stated has been often supported. There is no more difficulty, it has been argued, in conceiving how vital properties may be superadded to organized matter, than in understanding how magnetic properties may be communicated to iron: but the latter process appears to be really of a nature different from what is commonly supposed. The so-called communication of magnetic properties to iron is nothing more than the production of a change in the condition of the metal by which the electric properties, previously existing in that as in every form of matter, are manifested, and caused to give rise to magnetic powers. A little consideration will shew that we cannot become cognizant of any property of matter without some change being effected, either directly upon our organs of sense, or upon them through the medium of its action with some other material body. Every such change, therefore, requires certain conditions, without which the property cannot be manifested; and, until any form of matter has been placed in the conditions requisite to develope a particular property, we have no means of judging either of its presence or absence. It is perfectly correct to say that organized matter exhibits itself to the mind in a relation totally different from that of inorganic matter, and that properties apparently new are thereby manifested. But no one can assert that there does not exist, in every particle of matter, the capability of exhibiting vital actions, when placed in the requisite conditions; in other words, when made a part of a living system by the process of organization. It is only the complexity of the conditions required to manifest it which prevents our recognizing this capability as a common property of matter, or at least of those forms of it which we know by experience to enter into the composition of organized structures.

Of all the branches of science whose phenomena are dependent upon simple physical principles, that of Meteorology is the most obscure and apparently uncertain. Although the changes which become the subjects of observation are constantly occurring under our notice, the difficulty of controlling and artificially combining the agents concerned in them prevents us from deriving much assistance from experiment; whilst the complexity of the conditions under which similar effects occur, and the variety of results which may arise from very slight modifications of the same cause, render such a mode of enquiry peculiarly necessary.

When we turn from the inorganic world to contemplate the living kingdoms of nature, we at once perceive a vast difference in the objects of our investigation; and we may perhaps be led to suppose that this change requires that our mode of philosophising should be varied in accordance with it. But a little reflection must convince us that if the inductive system be really founded upon the relation between the human mind and the world in which man is placed, it must be as applicable to the discovery of general laws in one department as in another; and that, although each of the different steps may be individually modified according to the character of the objects upon which our reasoning is founded, the general plan of the whole must be the same, in whatever particular channel our labours are directed. We have it on record that Newton, after the noblest effort of human reason that philosophy has perpetuated, conceived the idea that the laws governing the structure and functions of living organisms might be discovered by the comparison of their similar organs and functions, as were those of the inorganic world by the study of the uniformity of its phenomena. ("Idemque dici possit de uniformitate illâ quæ est in corporibus animalium.") Until, however, the principles of philosophical induction are thoroughly understood, the peculiar combinations in which vital phenomena present themselves to our notice, their apparent dissimilarity from the changes which we witness in the world around, and their obvious adaptation to particular ends, might lead us astray into the labyrinth of unprofitable speculation with regard to the presiding agencies by which they are governed; and the slightest acquaintance with the history of physiology will convince us that this has been the case up to a very recent period, and that in fact the legitimate objects of investigation and the true mode of pursuing them are only now beginning to be understood.

We shall connect our views on the study of physiology as an inductive science, with some criticisms on Mr. Whewell's history of this branch of philosophy, on which, as we have before hinted, we cannot bestow the same approbation that is due to the portion of the work devoted to the physical sciences. The author has not attempted to give even a general sketch of the progress of the science, "as such," he says, "would suit neither my powers nor my purpose;" and in the following remarks, he explains more fully his grounds for avoiding the task.

"Like most other ancient sciences, its career has been one of perpetual though variable progress; and as in others, so in this, each step has implied those which had been previously made, and cannot be understood aright, except we understand them. Moreover, the steps of this advance have been very many and diverse; the cultivators of anatomy have in all ages been numerous and laborious; the subject is one of vast extent and complexity; almost every generation has added something to the current knowledge of its details; and the general speculations of physiologists have been subtle, bold, and learned. It must, therefore, be difficult or impossible for a person who has not studied the science with professional diligence and advantages, to form just judgments of the value of the discoveries of various ages and persons, and to arrange them in their due relation to each other. To this we may add, that, though all the discoveries which have been made with respect to particular functions or organizations are understood to be subordinate to one general science, the philosophy of life, yet the principles and doctrines of this science nowhere exist in a shape generally received and assented to among physiologists; and thus we have not, in this science, the advantage which in some others we have possessed, of discerning the true direction of its first movements, by knowing the point to which they ultimately tend; of running on beyond the earlier discoveries, and thus looking them in the face, and reading their true features." (Vol. iii. p. 379.)

Fully admitting that physiology has not advanced as a science to the same level with astronomy, or optics, or electricity, we cannot agree with Mr. Whewell as to the impossibility of truly estimating the discoveries and doctrines of past times. We certainly do not fully understand the value of insulated facts, until they are made the foundation of general laws; but we may have a clear perception of their importance in leading to such inferences, although from various retarding causes the laws themselves have not yet been clearly brought into view. Mr. Whewell has subsequently very justly remarked:

"We are by no means to confine ourselves to the positive discovery, and reject all the less clear and certain speculations. To do this would be to lose most of the chances of ulterior progress; for though it may be that our conceptions of the nature of organic life are not yet sufficiently precise and steady to become the guides to positive inductive truths, the only way in which these peculiar physiological ideas can be made more distinct and precise, and thus brought more nearly into a scientific form, is by this struggle with our ignorance or imperfect knowledge. This is the lesson we have learned from the history of physical astronomy and other sciences. We must strive to refer facts, which are known and understood, to higher principles, of which we cannot doubt the existence; and of which, in some degree, we can see the place, however dim and shadowy may be the glimpses we have hitherto been able to obtain of these forms. We may often fail in such attempts; but without the attempt we can never succeed."

With regard to the different physiological hypotheses, which have abounded from the time of Pythagoras to the present epoch, the remark we have already made respecting the various systems of philosophy is peculiarly applicable; each hypothesis was the consequence of a hasty generalization of a limited number of facts, and consequently was of use in bringing those facts prominently forward; so that whilst no one had a solid foundation, no one could be regarded as utterly futile. Now that it is universally acknowledged that physiology, like other sciences, must be built up of the materials supplied by observation only, and that the erection of the edifice must be preceded by the demolition of the ruins of the showy but unstable structures of former times, we cannot but consider ourselves quite competent to pronounce upon their merits, since the utility of each to the final consummation will be made apparent by the number and variety of the facts it has brought into view. Thus the chemical and mechanical physiologies of the seventeenth century have long since died a natural death; but each sect contributed much to the advance of our knowledge of the animal economy, and many important truths might be adduced in support of their propositions. The sect of the vitalists originally promulgated doctrines scarcely less erroneous than those to which we have just alluded; and although physiologists have not yet been able to agree in fixing the boundaries of physical and vital actions in the living body, there are none who do not allow that both mechanical forces and chemical powers are in constant and extensive operation. Nor do we feel any greater difficulty in estimating the merits of the system of Brown, who was certainly the first to give clear expression to the truth that the actions of life depend upon the excitement of vital properties by external stimuli; but while this important principle is made the foundation of modern physiology, the pathological system which its author connected with it, has disappeared almost as rapidly as it was raised. Surely, with regard to all these systems and doctrines, we are fully competent in the present day to pronounce an opinion; and as to those of more recent date, we do not apprehend that any difficulty will much longer exist, since the multitude of observers everywhere engaged in applying and testing them, will speedily disclose their true value.

Declining to treat of the general progress of physiology as an inductive science, Mr. Whewell has thought it sufficient "to trace the establishment of some of its more limited but certain doctrines;" and with this intention he has given a brief outline of the discovery of the connexion of nerves and muscles as organs of voluntary motion, by Galen and other ancient anatomists; and of the discovery of the circulation by Harvey and his predecessors. To these he subsequently adds a notice of the discovery of the motion of the chyle, and of some of the theories of digestion; an examination of the process of reproduction (his remark on which, "that it offers to us laws and principles which include both the animal and vegetable kingdoms," might with equal correctness be extended to every one of the organic functions), and a sketch of the discoveries respecting the nervous system from the time of Galen to those of Bichat,

Bell, and Mayo. None of these subjects are, we conceive, treated in such a manner as to give a correct idea of their real progress.

The principal error into which Mr. W. appears to us to have fallen, is one by no means peculiar to him; and we shall take the more pains to expose the fallacy, as clear ideas on this subject are peculiarly necessary to the distinct conception of physiology as an inductive science. The *doctrines,* as Mr. W. terms them, of the circulation of the blood, of the motion of the chyle, of the connexion between nerve and muscle, and of the functions of particular nerves, are not *laws* even of a low degree of generality, but *facts* on which general laws are to be founded. Physiology, it is to be recollected, is the science of life; not the knowledge of the functions of the human body only, but the accumulated and classified amount of all the changes occurring in living organized beings. If we were perfectly acquainted with the structure and functions of any one organism, we should have a collection of *facts* to be included with those derived from the study of other dissimilar organisms, in general expressions, which, to be of real value as the foundation of higher inductions, must be of *universal* application. Every living being, therefore, may be looked upon as a collection of phenomena, each of which may, when properly understood, contribute to the establishment of some general law governing either the development of the material structure or the functional changes to which that structure is subservient; and it is obvious that until these phenomena are correctly and certainly ascertained, they cannot be made available for any scientific purpose, although hypotheses may occasionally be advantageously erected upon them, the conformity of whose results with known facts may afford support to the original supposition. Now it is in the difficulty of correctly ascertaining facts and of satisfactorily observing phenomena, that the true obstacle to the progress of physiology as a science is to be found. Though the structure of the human body has been carefully and minutely examined by so many thousands of anatomists, how many points are still uncertain, and how much yet remains to be discovered! and yet this structure is but one of those groups of *instances,* which must be collected from the many hundred thousand species of plants and animals which the naturalist believes to exist on the surface of the globe, before we can have sufficient data for the establishment of the most comprehensive and universal laws regulating the development of living organisms.

The difficulties which present themselves in the observation of the facts which it is the object of the science of anatomy to ascertain and generalize, are as nothing to those which beset the path of the enquirer into the changes which living beings perform and undergo during the whole period of their existence. These may be practically divided into the normal and abnormal, the former class being usually designated physiological, the latter pathological. It is obvious that this use of the term physiological is much more restricted than the sense in which we have already employed it; and perhaps it would be convenient to confine it to its usual acceptation, and to avail ourselves of the term biology to signify the science of life in its most extended sense. It may be surmised, however, without improbability, that pathological as well as physiological phenomena are referrible to the ultimate vital properties of organized tissues; and that, instead of requiring a distinct set of laws for their explanation, they will prove to be results of these primary causes, as necessary as those perturbations of the planetary system which appear at first sight to interfere with the law of gravitation, but which a more enlarged conception of the application of that law includes amongst its inevitable consequences. The sum of all the phenomena which constitute the life of an organized being, is to be regarded as a collection of *facts*, of which each must be stated in a separate and concise form, before it can be made the subject of any general expression founded upon similar facts derived from the study of other living beings.

There are a few *laws* of a low degree of generality which might be deduced from the study of a single organism: thus the human physiologist might fairly infer that all muscular fibre possesses the property of irritability, because he is able to prove the existence of this property in every one of the numerous fibres which enter into the structure of the human body; and extending his views to the remainder of the animal kingdom, he would probably find that wherever muscular fibre exists, it possesses the same capability of contracting on the application of a stimulus. But were he to attempt to generalize still further, and to assert that every tissue manifesting this property possesses the characteristic structure of muscular fibre, (which tissue alone manifests it in man and the higher animals,) he would find himself widely astray from the truth; since a knowledge of the anatomy of vegetables and of the lowest animals shows that nothing analogous to the muscular structure exists in tissues which distinctly exhibit the property. We cannot, therefore, look for an explanation of this property in any law of the connexion of a particular function with a certain form of structure, which will not include all the tissues found to be possessed of it. Now the great difficulty in physiological investigation results from the complexity of the combinations in which vital phenomena present themselves, and their dependence upon one another to a degree that almost entirely precludes their separate examination. Were we able to ascertain *facts* regarding the changes which take place in the interior of the living body as easily as the astronomer observes the place of a planet, or the chemist the decomposition of a salt, there is no reason whatever to prevent these facts being generalized in the same manner and to the same degree with those of the physical sciences. The chemist, when desirous of ascertaining to which of the ingredients in a given mixture a particular effect is due, places each separately in the conditions required to produce the result; but the physiologist finds that the attempt to insulate any one organ, and reduce the changes performed by it to definite experimental investigation, necessarily takes away or considerably alters those very conditions under which alone its functions can be normally performed, and that he is totally unable to produce similar changes by new and artificial combinations of elementary materials. Frequently, too, the changes when they might otherwise be ascertainable, are of a kind imperceptible to our means of observation; and as we only know of these by the effects they produce on others, there must be considerable uncertainty with regard to their nature and even their very existence. Thus we only know of the nervous influence supposed to be transmitted along the motor nerves, by its effects in producing muscular contraction; as to the nature of these changes in the nervous matter in which the propagation of the influence consists, we cannot form even a probable guess; and the nervous influence supposed by some to be essential to the organic processes of nutrition, secretion, &c. is only suspected from the sympathy which evidently exists between these processes and certain states of the nervous system.

Another difficulty in the investigation of the phenomena of life arises from this; that whilst, in considering the objects and changes with which the inorganic world presents us, we are able to connect the external or evident characters of bodies with those properties which we know them by experience to possess, and thus to predicate the latter from the former, we are totally unable to follow the same course with regard to organized structures, owing to the extraordinary differences in the properties possessed by tissues, which, as far as our means of observation extend, present exactly the same external characters. Hence it is that we see so many dissimilar phenomena occurring under circumstances apparently the same, and so many changes of the same nature referrible to causes which would seem so different. It is this which has given to all the sciences of life a character of such great uncertainty: we cannot doubt that the laws of nature are as constantly regulating the changes to which living beings are liable, as those of dead matter; but from the apparent inapplicability of ascertained laws to novel cases, and the impossibility of accurately acquainting ourselves with the conditions upon which our application is founded, the physiologist and the medical practitioner are alike baffled when they attempt to predict even the most common phenomena as the result of their operation.

We cannot but perceive, therefore, that the difficulty of ascertaining *facts* in physiology obliges us frequently to adopt opinions and speculations as the foundations of our reasoning; and that these opinions are themselves open to peculiar sources of fallacy. Where, however, a fact regarding the connexion of a particular organ with a special function is definitely ascertained, it may fairly be classified with others of the same nature, so as to serve as the foundation of the inductive process; but care must be taken to include *all* the facts having a relation to the subject, in order that our induction may be really valid. It is by a process of this nature that the essential or elementary structure of all secreting organs is found to be a membrane minutely reticulated with blood-vessels and permeable to fluid; the peculiar arrangement of the surface of this membrane, its convolution into the tubular structure of glands, and the presence of a reservoir and excretory duct for the secreted fluid, being all matters of secondary importance not influencing the general result.

The mass of facts relating to the structure and changes of living organisms, which are collected by the sagacity and ingenuity of observers and experimental enquirers, must be classified and arranged before they can become subservient to the purposes of science; and this object is accomplished in different ways according to the laws of which the philosopher is in search. Thus the physiologist, whose aim it is to discover the ultimate vital properties of matter and the laws of their operation, considers the *individual* changes which in their totality make up the life of an organized being, and, arranging them into groups termed functions according to their similarity with each other and their obvious tendency to the same end, pursues his enquiry into the nature and exercise of each, through all the forms of organized beings in which it is manifested. The object of the naturalist, on the other hand, is to arrive at the laws which regulate the combination and structure of the organs on which the functions depend; and he therefore, viewing each organism in its *totality*, arranges similarly formed beings under the same group, placing as the character common to the whole the points in which they agree, and leaving the subordinate differences to be added to this common character, in order to express the qualities of an individual. This classification (resembling that of the physiological changes into functions) is but a step towards the establishment of general laws by which the structure of the organized kingdoms of nature is regulated. These laws, expressing the manner in which organs are combined and adapted to each other, the relative development or simplicity of each, the modifications which the primary types or elementary forms of each may undergo according to the circumstances in which the being is to be placed, and various other conditions of its formation, it is the object of the naturalist to ascertain; and any mode or system of classification which he may adopt is valuable in proportion as it keeps the establishment of these laws in view, and facilitates the accumulation of the knowledge upon which they must be founded. The connexion between these two branches of investigation is so intimate that neither can be pursued with probability of success, without a considerable knowledge of the data and principles upon which the other is founded; and he will evidently be the most likely to arrive at the discovery of important general truths in either, who includes the whole of the phenomena of life in one extensive survey. The physiologist refers to the naturalist for instances in which a function is performed on the same general plan, but under a great variety of circumstances, as manifested by the adaptation of the structure of the organ to the medium of existence, (e. g. the formation of the respiratory membrane into lungs or gills;) whilst the naturalist refers to the physiologist to assist him by the examination of the function and development of an organ in determining its real character, to which the consideration of its form and structure alone might not lead him. The natural system of botany affords a beautiful example of this kind of investigation: and there can be little doubt, from the advances recently made, that some of the most important laws, regulating the structure of living beings and the combination of their organs, will be speedily disclosed to view.

The objects of physiological enquiry are twofold: first, to ascertain the changes actually taking place in the living system; and second, to determine the conditions of those changes; and hence to arrive at the general laws of vital action. Both of these classes of enquiry we regard as principally dependent upon observation for their materials, and the inferences to be deduced from it are, we conceive, much more definite and certain than those which experiment affords; but it will be seen that we comprehend under the former term many kinds of investigation which are usually considered experimental, and that there are in physiology many circumstances which render a purely experimental enquiry less available than in any other branch of science.

The physiological observer, then, has for his first object to determine the nature of the series of changes of which the sum constitutes the life of any living system. His most simple and direct means of observation, namely, his unassisted senses, inform him of those obvious changes only which in some instances mark the relations between the being and the external world, and in others are to be regarded as the results of the processes taking place within the system. Thus a physiologist, content with simply watching the growth of a plant in its natural situation, would see little beyond its successive increase in size and the development of its different parts: its absorption of nutriment from the soil or the atmosphere would not, under ordinary circumstances, become apparent to him; and, in fact, the only obvious relations between the living system and the external world would be evinced by the influence of light and moisture upon the leaves and flowers. With regard to animals, simple observation would exhibit to him not only the results of the organic functions made evident by the increase and development of the bodily frame, but those changes which immediately connect them with the external world, the ingestion of aliment, and the discharge of excrementitious matter. He would also perceive the manifestations of the animal power of locomotion, and might infer from the analogy of his own actions that sensations were being produced, and were giving rise to mental processes. These, then, are all the means of information which unassisted observation can afford; we may conceive them to be such as brutes possess, and we can readily understand how small must be the real amount of knowledge to which they can lead.

But the ingenuity of man has enabled him to devise methods of adding greatly to this meager outline, by contriving methods of more exact observation, and of thus discovering what would otherwise be imperceptible to the senses. We will take for illustration one of the simplest possible of these aids. No one, on merely looking at a plant in active vegetation, would suppose that its leaves are constantly exhaling a large quantity of fluid from their tissue; but, by holding a polished surface in their neighbourhood, it is found speedily to become dimmed with moisture. This *observation*, then, (for it is nothing more,) leads to the knowledge of the function of exhalation. Again, a plant vegetating under common circumstances would not be suspected of constantly taking up a large quantity of fluid by its roots; but, if these be placed in a limited quantity of damp earth, so that the loss is made perceptible, the moisture will be found to disappear from the soil much faster than by ordinary evaporation. Here is another observation of the simplest kind bringing us to the knowledge of the function of absorption; and though in such a case the alteration in the conditions necessary for the observation might cause the process to be regarded as an *experimental* one, a little reflection will shew that the change is not in the body whose functions we are examining, (for we are anxious to preserve these unaltered,) but in the external conditions made favorable for our observations; just as the chemist, to observe the colour of a solution, would transfer it from an opaque vessel to a transparent one.

Having, then, arrived at the knowledge of these functions by simple additions to our ordinary means of observation, a further refinement of our methods will add to our acquaintance with them: thus, to continue our former illustration, the judicious employment of the balance will enable us to determine the quantity of fluid absorbed and transpired by a particular plant, and the proportion it bears to its surface. The researches of Hales on this subject, and, in like manner, those of the various physiologists who have investigated the gaseous changes produced by vegetables, have usually been denominated *experimental;* but it appears to us that the term is no more applicable to them than to the measurements of the altitudes and distances of the heavenly bodies, performed by the astronomer. It is true that, in the latter case, the objects are entirely beyond the reach of the observer, and his aim is to adapt his measurements and calculations to convey a correct idea of their true relations; whilst, in the former, the objects are certainly under his control, but he endeavours to preserve them as much as possible in their natural conditions, and simply to ascertain the changes they effect on the surrounding fluid or aëriform media, by measuring the quantities of these media which are lost or added. Whenever any change in the conditions of the living system takes place, (as when a plant becomes sickly from inclosure in a confined atmosphere,) the observation becomes valueless for the purpose it was intended to serve.

The physiological observer may employ other means of assisting his senses, by which he is enabled to detect changes inappreciable without such aid. Thus, he avails himself of the microscope for examining the capillary circulation, and endeavours to place the part employed for observation as nearly as possible in its natural relations; avoiding the influence of gravitation and of the loss of fluid from neighbouring divided vessels, both of which have an evident effect on the movement of the nutritious fluid, in plants as well as animals. Other means of magnifying changes of various kinds, which would be otherwise inappreciable, might be easily referred to: thus, the use of the thermo-electric multiplier has enabled MM. Becquerel and Breschet to detect minute alterations of temperature during muscular action; in which observation it was requisite to guard against various errors arising from change in the bodily condition, which might have interfered with the correctness of the result; and, according to the statement of Mr. King,* multiplication of insensible motions by means of a lever enabled him to detect venous pulsation, even after allowing for the disturbance which might be occasioned by a neighbouring artery. We speak of all these as observations rather than experiments, because their object is to ascertain the natural changes going on in the living body under their normal conditions; and any means we may employ of rendering them perceptible to the senses are just as independent of the changes themselves as are the preparations made by an astronomer to view and measure the heavenly bodies, by erecting his telescope, and rendering it fit for use by uncovering its glasses. As soon as our methods of observation do affect the conditions of the change, the result is attended with more or less of uncertainty, in proportion as we are ignorant or aware of the extent and consequences of the influence produced.

But the physiological observer does not confine himself to the simple collection of phenomena externally presented to his notice, and which are often, in fact, the mere results of those which take place in the living system: he endeavours, by an examination of the interior of that system when in a state of activity, to ascertain the nature of those on which the former are dependent. He will be more or less successful in discovering

* Guy's Hospital Reports, vol. ii. p 108.

these, in the inverse proportion to the derangement of the train of vital actions which shall have been created by the violence he is compelled to inflict; since it is evident that, in the examination of the normal changes of any one part, it is essential that the functions of the whole system shall go on with as little disturbance as possible, owing to that mutual dependence of which we have already spoken as one of the greatest obstacles to physiological enquiry. It is in this manner that the absorption of the chyle, the motions of the heart and alimentary canal, and various other interior phenomena of a similar kind, have been discovered; and we think it quite evident that such researches cannot be denominated *experimental*, since no new conditions have been brought into action, but the original ones are preserved as far as circumstances will permit.

We have already remarked that there is a series of changes in the living body, with which no means of observation that we at present possess can make us directly acquainted. We, of course, allude to those which take place in the nervous system. No one doubts that when a stimulus, either external or internal, excites contraction in a voluntary muscle, it is through the medium of an organic change of some sort, propagated along the nervous cords. But, if we examine the foundation of this opinion, we shall not find it in any phenomena with which the normal condition of the system presents us: it is derived by inference from *experiment;* that is to say, a change in the relative conditions of nerve and muscle, effected by intercepting the influence supposed to be propagated along the former, or by exciting a new influence by various stimuli applied directly to itself. We might, for the sake of argument, object that these data are insufficient to afford a positive conclusion, since the wound necessary for the division of the nerve might of itself produce the effect: it would be easy, however, to shew, by a parallel experiment, that this is not the case in the present instance. Still we could only positively infer the transmission of nervous influence, by causing it to act upon a part to which it previously was not conducted; an experiment obviously beyond our power to perform, except by bringing together the two ends of a divided nerve. In another case, moreover, the impossibility of obtaining such a positive inference leaves us in uncertainty if we trust to experiment alone: we refer to the question of the innervation supposed by some to be conveyed to the organs of nutrition and secretion. Here the complications of the experiment are so great, and so liable to affect the result, that we very much doubt if any satisfactory conclusions can ever be drawn from it.

It will be seen, then, that our knowledge of the changes occurring in the living system is founded upon *observation* only; except where we cannot take cognizance of the changes themselves, and can only infer their existence from the inaction which ensues when the organs to which we attribute them are abstracted or insulated. We shall now enquire what is the most legitimate mode of pursuing the second branch of physiological enquiry,—the conditions of these changes.

It is obvious that experiment will be here more available; but even here judicious observation may prove, in a majority of cases, an effective substitute for it. The first subdivision of this investigation will evidently be the examination of the dependence of the vital functions upon external agents; a subject on which the researches of Dr. Edwards have shed so much light. Yet we do not call the researches of Dr. Edwards entirely experimental, since his aim was, in many instances, to determine the regular sequence of the vital functions, in conformity with the usual changes of age, reason, &c. They were experimental whenever the animals were placed in entirely new conditions, and means taken to observe the influence of those conditions; and it was in these cases that the greatest care was necessary to guard against fallacy in the results, by taking the average of a large number of instances. It is in experiments of this kind that we can most successfully imitate the chemist or mechanical philosopher, in testing the conditions necessary to a particular result, by excluding each in succession; as when Dr. E. shewed that the presence of oxygen in the atmosphere is not immediately essential to the excretion of carbonic acid from the lungs; and that the want of alteration usually perceived in the quantity of nitrogen in respired air is due to the equality between its absorption and exhalation. There are some instances in which the same mode of enquiry is applicable to the conditions of internal changes; as when the first sound of the heart was shewn to depend principally upon muscular action, by the successive exclusion of all the other suspected causes.* But, in this case, it is to be observed that the effect whose conditions were to be examined was a merely physical one, and that these conditions could be closely imitated by artificial means.

Far different is it with regard to the conditions of the purely vital functions. As we have before remarked, the attempt to insulate any of these, and make it the subject of special examination, destroys at once the capability of its performance; and we cannot imitate it by any new combinations of matter. Hence experiment can conduct us very little farther in this enquiry than the determination of the dependence of the functions upon one another; and we believe that all which it has yet detected (especially regarding the functions of the nervous system,) may be reduced to this or the former place in our classification. And, as we have before hinted, many of the results obtained from it are liable to great uncertainty, from the general functional derangement which almost inevitably attends the performance of the necessary operations, arising out of the closeness of that bond of union which links together, in the higher animals especially, all the changes concerned in the maintenance of the vitality of the system. In the lower classes of animals, where this connexion is much less decided, and in vegetables, where it is still looser, we can insulate particular organs, and study the conditions of their actions to a much greater extent; and hence these are the most favorable subjects of experimental research on the organic functions.

But the physiologist is fortunately not confined within these limits: the ever-varying forms which the study of animated nature submits to his observation present him with manifold combinations of causes and concurrents, whilst the phenomena they exhibit shew him the results of these combinations. He may, therefore, advantageously avail himself of them for the purpose of ascertaining what are the conditions essential to the occurrence of any particular change, and what are accidental, or at most have a secondary influence upon it. The chief obstacle to his progress in this mode of investigation arises from the difficulty of correctly observing the phenomena of whose causes or laws he is in search; since the very change

* See vol. ii. p. 598, and Transactions of British Association, vol. v.

of circumstances which may be essential for observation would be liable to derange the train of vital actions, which it is his object to observe in their normal state. Moreover, he is perplexed by the immense number and variety of the phenomena which are thus presented to his notice, and the dissimilarity of the effects which a very slight, in some instances an inappreciable, change in the conditions produces. This perplexity, however, is of the nature of the *embarras des richesses;* and we agree with the observation which has recently been made, "that it must be the fault of our mode of study if we do not arrive at some tolerably definite conclusions" from these most abundant data.

Respecting the method in which these data are to be classified, when once accurately substantiated by observation, we have already entered into some detail; and we shall now conclude the subject by one or two illustrations of what we regard as the superiority of observation over experiment, in elucidating disputed and important questions in physiology.

It is well known that a controversy has long existed regarding the dependence of the capillary circulation on the heart's action; and the attempts made to determine it by experiment have never been satisfactory to either party. We shall see that we may easily arrive at a definite result by observation only. In vegetables, the vital circulation (that of the elaborated sap*) is entirely capillary, and is kept up without any central organ of impulsion; and observation of the influence of light, heat, &c. on the development of the foliaceous organs proves that the afflux of crude sap to them in the spring is the effect of the vital processes there performed. In the lowest of the animal kingdom possessed of a distinct circulation, such as the Echinoderma, the system is destitute of a central organ of impulsion; and we find this evolved in ascending to the articulated and molluscous tribes. It is difficult to observe the circulation in insects, or in such transparent Entomostraca as the Monoculus Pulex, without coming to the conclusion that, though a contractile cavity exists, the movement of the fluid is mainly independent of its pulsations.† The higher we ascend in the animal scale, the more do we observe the principle of centralization in its functions becoming apparent; and accordingly, in the Vertebrata, we find the power of maintaining the circulation less diffused through the system, and more concentrated in the heart. Still, however, various phenomena indicate, to those not indisposed to appreciate them, that capillary action has an independent influence, although its nature may not yet be understood; and that, even in man, the circulation is not altogether dependent upon the heart.

A very analogous question has long been agitated regarding the dependence of the organic functions upon the ganglionic system of nerves. The obstacles attending any endeavour to determine it by experiment are too obvious to need indication; but we shall briefly point out what knowledge we may attain from observation. In vegetables, we have an example of the performance of all the organic functions without anything analogous to nervous influence; since all attempts to prove the possession by them of a nervous system, under any form, have signally failed; and these

* See vol. iv. p. 27.

† It appears not improbable that the non-development of a distinct organ of impulsion, so remarkable in the articulated classes as compared with the Mollusca, may be the natural counterpoise to the high degree of capillary power (if we may be allowed the expression,) which is created by the very active nutritive processes of these classes.

attempts were only excited by an indisposition (the obvious remnant of former dogmas) to admit the possibility of any vital actions being independent of this system. As to the existence of a nervous system in the lowest animals, we shall not make any positive statements, since the difficulties which attend the minute examination of their tissues are much greater than those which the vegetable anatomist encounters. The simplest form of nervous system which we meet with appears to be connected rather with the locomotive than the nutritive apparatus; and it is now universally admitted that the double nervous cord of the Articulata, and the cyclo-ganglionic system of the Mollusca, represent the cerebro-spinal system of the Vertebrata; and that these classes possess the separate rudiments of a distinct visceral system of nerves. Believing that the office of the latter is to harmonize and connect the different organic functions more completely than can be effected by the circulating apparatus, which is their only bond of union in plants, and to bring them into relation also with the functions of animal life, we should expect to find it developed in proportion to the general concentration and specialization of the functions, and to the development and predominance of the animality; and so accordingly it is. If, on the other hand, the organic functions had been *dependent* upon it, we should have expected that it should have been developed according to their predominance; which observation absolutely contradicts.

We must now return to Mr. Whewell's book, from which we shall draw one or two illustrations of the views which we have been propounding. By classing the knowledge of the circulation of the blood, of the motion of the chyle, &c. among "the more limited but certain doctrines of physiology," Mr. W. appears to us wanting in "clear ideas" as to the true import of these discoveries. To those who have followed us through the preceding remarks, we think it will be evident that the knowledge of the circulation of the blood in man holds just the same rank in physiology as the fact that a stone unsupported falls to the ground, in general physics. In its practical applications, the former fact is of the highest importance; but, as contributing to the establishment of any general law, it is valueless until combined with others. We shall suppose that Harvey, who established the existence of a general circulation in the higher animals, and Hales, who so ingeniously investigated the same function in plants, had thence inferred that circulation was a function common to all organized beings, and necessary to their existence,—they would have committed the common error of generalizing falsely upon a limited induction; for a more extensive acquaintance with comparative anatomy and physiology would have informed them that the object of the circulation being merely to convey the absorbed nutriment to the respiratory organs and to the tissues it is to maintain, there is no occasion for any motion of fluid in those beings (some of which occur both in the animal and vegetable kingdoms,) which absorb equally from the whole surface, and which possess no special respiratory apparatus. In fact, the development of the circulating system, and the complexity of its function, bear a strict relation with the degree of specialization of the absorbent surface, or, in other words, with the confinement of the absorbent power to some particular part of the general surface, or of its internal or external prolongations.

Mr. Whewell has very properly cited the laws of vegetable morphology as illustrative of the generalization of which physiological facts are susceptible; and he justly remarks, that the terms "*metamorphosis* and *development*, which are here employed, convey ideas entirely different from any of those to which the physical sciences have led us in our previous survey; and are, in short, genuine *organical* or *physiological* ideas; real elements of the philosophy of life." There is certainly no science which has more need of definite terms than physiology, involving as it does so many abstract ideas and speculative opinions; and there is assuredly none which has been more retarded by the variety of senses which have been attached to the same word by different authors. It is much to be desired that the explorers of this important and extensive domain, could fix upon a set of definite land-marks, by the aid of which they could render their discoveries and prospects intelligible to each other and to their respective followers. What the present state of chemistry would have been without a settled nomenclature might not be altogether an unprofitable speculation; and although we do not consider that such a reform is needed in physiology as that which Lavoisier effected for chemistry, it is particularly desirable that there should be no room for hesitation as to the meaning to be attached to commonly received terms in any particular instance.

The last chapter of Mr. Whewell's work consists of an exposition of the author's views on the employment of final causes in physiological research; and we are surprised to find that he considers the philosophical pursuit of physiology as inconsistent with the recognition of their value. But surely, whilst the study of final causes is of great value in leading to the *discovery of facts*, the search after general laws to be based on these facts must be totally independent of them; and the higher we advance in the attainment of generalizations, the more exalted our ideas necessarily become of that designing mind which planned and adapted the organized as well as the inorganic creation. The vagueness of Mr. Whewell's ideas regarding the nature of physiological discoveries, has pervaded his opinions on the use of final causes; and he has consequently looked with a suspicious eye upon all attempts to philosophise independently of them. Now we shall quote, from no less an authority in physical science than Mr. W. himself, what we regard as a very proper estimate of their value in that department of investigation; and we confess that we cannot discover any distinct line between physics and physiology, that necessitates such a variation in the mode of research.

"Final causes are to be excluded *from physical enquiry*; that is, we are not to assume that we know the objects of the Creator's design, and put this assumed purpose in the place of a physical cause. We are not to think it a sufficient account of the clouds that they are for watering the earth, (to take Bacon's examples,) or "that the solidness of the earth is for the station and mansion of living creatures." The physical philosopher has it for his business to trace clouds to the laws of evaporation and condensation; to determine the magnitude and mode of action of the forces of cohesion and crystallization, by which the materials of the earth are made solid and firm. This he does, making no use of the notion of final causes: *and it is precisely because he has thus established his theories independently of any assumption of an end, that the end, when after all it returns upon him and cannot be evaded, becomes an irresistible evidence of an intelligent legislator.* He finds that the effects, of which the use is obvious, are produced by the most simple and comprehensive laws; and when he has obtained this view, he is struck with the beauty of the means, by the refined and skilful manner in which the useful effects are brought about; points different from those to which his researches were directed." (*Bridgwater Treatise*, p. 353.)

It appears from Mr. Whewell's History of Physiology that he regards the adaptation of means to ends as so prominent a characteristic of organized beings that he considers no investigation into the laws of their structure can hope to be successful which does not keep this doctrine steadily in view. But we would ask him, with all possible respect, whether the object of his Bridgwater Treatise was not to demonstrate the harmony of means and ends in the structure of the universe, and thence to prove the existence of a designing mind? He has successfully shown that this adaptation is not less complete than that of the parts of a single organized being to one another. And would he not totally disregard, notwithstanding, the study of final causes in seeking for the laws of which that adaptation is the necessary consequence, although the observation of the facts upon which he establishes these laws might have been suggested, and the phenomena themselves brought to light by the perception of this general harmony and adaptation? We cannot point to a clearer instance of the necessity of a similar course in physiological investigation than that afforded by the generalization which has been effected in vegetable morphology, the laws of which Mr. Whewell allows to be firmly established and recognized. What would have been the situation of this science at the present moment, if the philosophic botanist had adopted the final cause or function of the several parts of the flower as his guide in investigating the laws of their structure, instead of tracing that structure through all its regular and irregular forms with a total disregard of its function? In considering the laws of the vegetable kingdom, we have abundant opportunities of observing how diversified, both in their forms and uses, are the various types which the same rudiments may assume; and that even when undeveloped, these rudiments appear as the necessary results of these laws, and assist man in the attainment and comprehension of them. No one, therefore, ought to be presumptuous enough to affirm that, though he has discovered an evident purpose in a particular structure, there may not be some other, less obvious, but really more important; nor, when he is altogether at fault as to the design for which some apparently useless part may have been created, has he any right to say that no object was to be fulfilled by it.

We cannot, therefore, see any ground for the indignation with which Mr. W. regards the speculations of M. Geoffroy St. Hilaire, on account of their neglect of what he seems to consider the *end* of physiological research, rather than the *means*. We do not pretend to know what may be the private opinions of that author with regard to the wisdom and power of the Creator; but we do assert, that the legitimate idea conveyed by his expression, "I ascribe no intention to God, for I mistrust the feeble powers of my reason—I observe facts merely, and go no further," has no more irreligious tendency than the passage we have quoted above from Mr. W.'s Bridgwater Treatise. We go further, and assert that the higher and more general the laws regulating the structure of animals which the physiologist can attain, the more will the contemplative mind be struck with the vastness of that designing Mind, which, in originally ordaining them, could produce such harmony and adaptation amongst their innumerable results. To use another very forcible expression of Mr. Whewell's, "the notion of design and end is transferred by the researches of science, not from the domain of our knowledge to that of our ignorance, but merely from the region of facts to that of laws."

To avoid all chance of being misunderstood in these views, we think it will be sufficient to adduce, in illustration of them, one of the most obvious and simple adaptations everywhere presented in the structure of animals,—that of the muscles to the skeleton. We constantly find, in pursuing our anatomical enquiries, that, for the advantageous attachment of muscles to bones, some particular form of the latter is provided; and that, where much power or a particular direction is required, a considerable prominence is given to the point of attachment. The teleologist would say, with apparent truth, that each of the bony processes was intended for the attachment of a muscle; and would thus be led to infer the form and direction of certain muscles of extinct animals, from the prominences which existed on their bones. He might go further, and say that the formation of the prominence is occasioned by the existence of the muscle; and might allege, in support of his view, the well-known fact that the osseous points of attachment are strongly developed in those persons who have much exercised their muscular system. On the other hand, the philosophic anatomist, fully acknowledging the adaptation between the osseous and muscular systems, would disregard it for the time, whilst seeking for the laws regulating the development of these systems; which laws he would seek to deduce from the observation of all the forms of each, both normal and abnormal. Thus, he would find that each of the important processes in the human skeleton exists as a separate bone in some of the inferior animals; and that the complicated muscular system of man gradually simplifies itself as he descends towards less specialized organisms. Supposing that the physiologist has succeeded in establishing such laws "independently of any assumption of an end, that end, when after all it returns upon him, and cannot be evaded, becomes an irresistible evidence of an intelligent Legislator." For we would fearlessly ask the opinion of any candid and reflecting person, whether it does not imply a far higher degree of Creative Wisdom and Power to suppose that, in the establishment of the laws of osteology and myology, (themselves probably subordinate to some higher principle,) all the results of each were foreseen and harmonized, so that every muscle, developed in accordance with the laws of *its* system, should find an attachment in the osseous process resulting from the action of the laws of *its* system,—than that the formation and adaptation of each separate muscle and each individual process required a distinct effort of creative skill?

We shall bring this subject to a close with a few observations on the most advantageous mode of studying the science whose present state and prospects we have been considering. It is but too true, that, in consequence of the limited amount of physiological knowledge which it has been hitherto thought necessary to communicate in most of the medical institutions of this country, few but university graduates have acquired any comprehensive view of the science; and of these no small proportion have been satisfied with receiving the information imparted to them, and retaining those portions only which they felt to be interesting or believed to be practically important. Recent changes, made in some of the metropolitan schools, encourage the hope that physiology will now rank no longer as an unimportant and therefore neglected branch of medical education, and that the knowledge of the functions of the human body in health will henceforth be regarded as a necessary preliminary to the investigation of the changes which it undergoes in disease. To the student who seeks merely to acquaint himself with what is already known in this branch of science, we have little advice to offer; since the perusal of the numerous works to which he has ready access, will afford him as much knowledge as he will remember, and a great deal more than he will be likely to profit by. But we hope that all of the rising generation are not of that class; and that there may be many amongst them earnestly desirous of aiding in the advance of a science unsurpassed in interest, scarcely rivalled in importance, and almost illimitable in the extent of the field it presents for cultivation.

It is quite evident that no one can advantageously commence the study of physiology without a tolerably complete knowledge of human anatomy, both general and special. Those details, however, which are peculiarly connected with physiological inference may perhaps be not improperly deferred until the time when their application tends to implant them on the memory. Either conjointly with, or subsequently to, the study of the human organism, we recommend that a general knowledge of comparative anatomy be acquired; and though the magnitude of the task may alarm the student, he will find that if he avoids devoting much attention to details of external form, and endeavours to make himself acquainted with the general development of each system, the pursuit will be easy as well as delightful. It will not be amiss to acquire at the same time a knowledge of the structure of vegetables; not only because we find there expressed in another and frequently a simpler form, the anatomical facts which it is difficult to trace in animals, but because the attainment of the laws of morphology in flowering plants, and their progressive extension to the cryptogamia, may advantageously serve as our guide in the more intricate pursuit of similar generalizations in the animal kingdom. We have already stated our belief that a knowledge of the principles of general physics is essential to the successful cultivation of physiological science; and when all these preparatory steps have been taken, the student will enter upon its study with no small advantages.

Whatever may be thought of the expediency of commencing the study of anatomy by investigating the structure of the simplest organisms, a plan which has many advocates, we are decidedly of opinion that this course is essential in physiology, and that the student who adopts it will be saved the necessity of unlearning many erroneous notions which he would unavoidably imbibe from the premature study of the human functions. In the pursuit of general physiology he will learn what are the essential conditions of life; he will see the changes indispensable to its support manifested in their simplest circumstances; and he will be able to ascertain what structures are necessary to their performance, and what additions and modifications these may undergo to suit the various purposes of their existence. He will acquire, also, the great advantage of making observation a substitute for experiment; the former means, wherever it can be employed in physiology being decidedly preferable, (as we hope we have successfully demonstrated,) both in the certainty and satisfactory nature of the conclusions which may be drawn from it, and in its freedom from those objections which every humane mind must feel to the infliction of unnecessary tortures upon beings endowed with sensations as acute as our own.

We have alluded, in the early part of this article, to the difficulty of distinguishing the operation of vital and physical laws; and this we cannot but regard as a question to be completely determined before the laws of purely vital phenomena can be satisfactorily established. To analyze the phenomena in which physical laws are acting under conditions supplied by vital processes, and to trace the diversities from their usual mode of action occasioned by the existence of those conditions, appears to us therefore to be at present the most obvious method of advancing the science. We cannot but believe that the enquiry would ultimately terminate in referring all vital actions to properties as essentially connected with that form of matter which we call organized, as are the ordinary physical properties with inorganic matter.

One more question would then remain: is it possible that the physical and vital properties of matter, which are at present our ultimate facts or axioms, may be hereafter included within a more general expression common to both? On this subject we can only speculate; but the probability appears decidedly in the affirmative. We have already remarked upon the rapid progress of generalization in the physical sciences, rendering it probable that before long one simple formula shall comprehend all the phenomena of the inorganic world; and it is not perhaps too much to hope for a corresponding simplification in the laws of the organized creation, although this is necessarily retarded by the many obstacles which the nature of the subject presents to the philosophical enquirer. In proportion to our attainment of such generalizations, we rise from the domain of our ignorance to that of our knowledge; for, at every successive step, we are able to comprehend new relations between facts that previously seemed confused and insulated; new objects for what formerly appeared destitute of utility.

Every step, then, which we take in the path of generalization must increase our admiration of the beauty of the adaptation, and the harmony of the action of the laws we discover; a beauty and harmony in which the contemplative mind delights to recognize the wisdom and beneficence of the Divine Author of the universe. If we can conceive that the same Almighty *fiat* which created matter out of nothing, impressed upon it one simple law, which should regulate the association of its masses into systems of almost illimitable extent, controlling their movements, fixing the times of the commencement and cessation of each world, and balancing against each other the perturbing influences to which its own actions give rise,—should be the cause not only of the general uniformity but of the particular variety of their conditions, governing the changes in the form and structure of each individual globe protracted through an existence of countless centuries, and adjusting the alternation of "seasons and times, and months and years,"—should people all these worlds with living beings of endless diversity of nature, providing for their support, their happiness, their mutual reliance, ordaining their constant decay and succession not merely as individuals but as races, and adapting them in every minute particular to the conditions of their dwelling,—and should harmonize and blend together all the innumerable multitude of these actions, making their very perturbations sources of new powers;—when our knowledge is sufficiently advanced to comprehend these things, then shall we be led to a far higher and nobler conception of the Divine Mind than we have at present the means of forming.

